# A risk-associated *Active* transcriptome phenotype expressed by histologically normal human breast tissue and linked to a pro-tumorigenic adipocyte population

**DOI:** 10.1186/s13058-020-01322-6

**Published:** 2020-07-31

**Authors:** Taekyu Kang, Christina Yau, Christopher K. Wong, John Z. Sanborn, Yulia Newton, Charlie Vaske, Stephen C. Benz, Gregor Krings, Roman Camarda, Jill E. Henry, Josh Stuart, Mark Powell, Christopher C. Benz

**Affiliations:** 1 Buck Institute for Research on Aging, 8001 Redwood Blvd., Novato, CA 94945 USA; 2 University of California, Genomics Institute, Santa Cruz, CA USA; 3 ImmunityBio, Santa Cruz, CA USA; 4 University of California, San Francisco, CA USA; 5 Susan G. Komen Tissue Bank at the Indiana University Simon Cancer Center, Indianapolis, IN USA

**Keywords:** Risk-associated normal breast tissue, *Active* transcriptome, Activated adipocytes

## Abstract

**Background:**

Previous studies have identified and validated a risk-associated *Active* transcriptome phenotype commonly expressed in the cancer-adjacent and histologically normal epithelium, stroma, and adipose containing peritumor microenvironment of clinically established invasive breast cancers, conferring a 2.5- to 3-fold later risk of dying from recurrent breast cancer. Expression of this *Active* transcriptome phenotype has not yet been evaluated in normal breast tissue samples unassociated with any benign or malignant lesions; however, it has been associated with increased peritumor adipocyte composition.

**Methods:**

Detailed histologic and transcriptomic (RNAseq) analyses were performed on normal breast biopsy samples from 151 healthy, parous, non-obese (mean BMI = 29.60 ± 7.92) women, ages 27–66 who donated core breast biopsy samples to the Komen Tissue Bank, and whose average breast cancer risk estimate (Gail score) at the time of biopsy (1.27 ± 1.34) would not qualify them for endocrine prevention therapy.

**Results:**

Full genome RNA sequencing (RNAseq) identified 52% (78/151) of these normal breast samples as expressing the *Active* breast phenotype. While *Active* signature genes were found to be most variably expressed in mammary adipocytes, donors with the *Active* phenotype had no difference in BMI but significantly higher Gail scores (1.46 vs. 1.18; *p* = 0.007). *Active* breast samples possessed 1.6-fold more (~ 80%) adipocyte nuclei, larger cross-sectional adipocyte areas (*p* < 0.01), and 0.5-fold fewer stromal and epithelial cell nuclei (*p* < 1e−6). Infrequent low-level expression of cancer gene hotspot mutations was detected but not enriched in the *Active* breast samples. *Active* samples were enriched in gene sets associated with adipogenesis and fat metabolism (FDR *q* ≤ 10%), higher signature scores for cAMP-dependent lipolysis known to drive breast cancer progression, white adipose tissue browning (Wilcoxon *p* < 0.01), and genes associated with adipocyte activation (leptin, adiponectin) and remodeling (CAV1, BNIP3), adipokine growth factors (IGF-1, FGF2), and pro-inflammatory fat signaling (IKBKG, CCL13).

**Conclusions:**

The risk-associated *Active* transcriptome phenotype first identified in cancer-adjacent breast tissues also occurs commonly in healthy women without breast disease who do not qualify for breast cancer chemoprevention, and independently of breast expressed cancer-associated mutations. The risk-associated *Active* phenotype appears driven by a pro-tumorigenic adipocyte microenvironment that can predate breast cancer development.

## Background

Previous studies of normal-appearing cancer-adjacent breast tissue have identified at least two different transcriptional phenotypes [1, 2], including an *Active* transcriptome phenotype associated with near 3-fold excess risk of future death from breast cancer [3, 4]. The histologically normal but molecularly altered nature of cancer-adjacent tissue has long been recognized and was originally referred to as “field cancerization.” More recently characterized by gene expression studies, this peritumor tissue microenvironment was thought to be unlike that found in normal healthy tissues and able to promote the growth and invasiveness of diverse tumor types due to its enrichment in wound healing, hypoxia, epithelial-mesenchymal transition, and pro-inflammatory gene signals [5]. While histologically similar to other peritumor microenvironments, cancer-adjacent normal breast samples were also thought to be transcriptionally distinct from reduction mammoplasty breast samples [1]. A microarray study of 79 non-malignant breast biopsies prompted by mammography, interrogating over 9000 variably expressed genes, identified two normal breast tissue subtypes including one that shared gene expression features with stromal, stem and mesenchymal cells [2]. Curiously, the biopsied females possessing these different transcriptome phenotypes did not significantly differ by age, BMI (body mass index), or mammographic density at the time of breast biopsy. Investigators therefore concluded that additional studies were needed to further characterize the cellular and molecular biology underlying the two different transcriptome phenotypes and to clarify their potential links to future breast cancer development [2].

Focusing on observed transcriptional differences among histologically normal cancer-adjacent breast tissues, Roman-Perez et al. first described a multi-gene (> 3700) signature capable of distinguishing normal breast samples expressing either an *Active* or *Inactive* transcriptome phenotype, the former characterized by increased expression of genes linked to cell motility, inflammation, fibrosis, and chemotaxis as well as decreased expression of cell adhesion, differentiation, and cell-cell contact genes [3]. While these two normal breast transcriptome phenotypes were not associated with any specific hormonal or intrinsic subtypes with respect to their adjacent breast tumors, patients bearing the *Active* peritumor microenvironment were 2.5-fold more likely to relapse and die of estrogen receptor (ER)-positive breast cancer over the next decade [3]. Using this same multi-gene classifying signature to interrogate cancer-adjacent normal breast samples acquired by The Cancer Genome Atlas (TCGA) program, Troester et al. identified 40% of samples with the *Active* transcriptome phenotype and showed in multivariate analysis that ER-positive patients with this peritumor microenvironment were 3-fold more likely to die of breast cancer within the next 10 years [4]. This last TCGA study made the surprising additional observation that histologically normal samples expressing the *Active* phenotype were composed of significantly more fat (mean 85% vs. 70%, *p* = 8.8e−05), fewer stromal (mean 8% vs. 19%, *p* = 0.00013), and epithelial (mean 7% vs. 9%, *p* = 0.027) cells, suggesting that the excess breast cancer mortality risk associated with the *Active* peritumor phenotype might be due to its adipocyte composition [4].

To explore the possibility that the breast cancer risk-associated *Active* transcriptome phenotype might actually be a pre-existent condition within an otherwise healthy-appearing woman’s breast tissue, and not simply induced by a nearby breast neoplasm, we studied the transcriptional phenotypes of normal breast biopsies donated by healthy parous women with no history of breast disease or specific mammographic abnormalities. Given the TCGA association of this *Active* phenotype with increased mammary fat content [4], we also examined the possibility that altered normal breast adipocyte populations contribute to this risk-associated *Active* transcriptome phenotype.

## Methods

### Donor population, breast biopsies, and histologic analyses

Paraffin-archived normal breast tissue samples were obtained from 151 parous, non-Hispanic white women (ages 27–66), without recent mammographic abnormality or history of breast cancer, who provided upper outer quadrant core biopsies for research purposes collected and archived by the Susan G. Komen Tissue Bank (KTB; Indiana University Simon Cancer Center, Indianapolis IN 46202). Donors supplied written informed consent and were recruited under a protocol approved by the Indiana University Institutional Review Board, and completed questionnaires with reproductive histories sufficient to calculate Gail 5-year risk scores (for all except 19 who were < age 35). Core biopsies were processed by standardized KTB operating protocols (KTB SOP) [6]; however, during our transcriptome analysis of these samples, we learned from KTB that the first 96 biopsy samples had been obtained and processed by 10% formalin fixation and paraffin embedding (FFPE), after which a change in the KTB SOP protocol occurred such that the next 55 sequentially obtained biopsies were processed by a formalin- and crosslinking-free PAXgene tissue preservation system (PreAnalytiX-Qiagen/BD, Switzerland) prior to paraffin embedding (PFPE). Given the two different tissue processing protocols, transcriptome analyses and donor characteristics were first performed and compared in a batch-specific manner (FFPE, F = 96 samples; PFPE, P = 55 samples); subsequently, an effort to batch correct and integrate all the gene expression data was also undertaken (see below). KTB provided digitized hematoxylin and eosin (H&E)-stained sections for every sample, from which cell composition was scored (% nuclei and area for fat, stromal and epithelial content, with additional comment about extent of leukocyte infiltration) by a dedicated breast pathologist blinded to all donor and batch details. Fat, stromal, and epithelial areas were quantitated using Aperio Image Scope software (version 12.3.2.8013, Leica Biosystems, Buffalo Grove, IL). This histologic analysis confirmed that no sample contained preneoplastic or neoplastic cells. Furthermore, terminal duct lobular unit (TDLU) counts were independently and blindly determined on each H&E slide as recently reported [7]. For adipocyte cross-sectional area assessment, four representative images from each H&E slide were analyzed at × 50 magnification (measuring ≥ 50 adipocytes per image) using Fiji imaging software with the open-source Adiposoft v1.13 plugin, as previously described [8].

### RNA sequencing for gene expression and gene signature scoring

Contiguous thick paraffin sections (10 μm each) sufficient to extract ~ 100 ng of total breast RNA per sample were sent to NantOmics, LLC (Culver City, CA 90232) for full transcriptome ribo-deplete RNA sequencing (RNAseq), performed on the Illumina NovaSeq platform. Reads were aligned using Bowtie2 v2.2.6 and RSEM v1.2.25 to RefSeq build 73 on hg19, generating both untransformed and log2-scaled TPM values for all expressed genes, publically deposited at https://xenabrowser.net/datapages/?cohort=Normal%20Breast%20(Benz%202020). Normalized, median centered, log2-scaled TPM values from the 96 F and 55 P batch samples were independently interrogated by unsupervised hierarchical clustering using 1318 variably expressed genes (IQR > 0.8) mapped onto our dataset from the > 3500 previously validated *Active*/*Inactive* classifying genes (see Supplement Table 1) to assign samples within each batch as having either the *Active* or *Inactive* transcriptome phenotype. This *Active* vs. *Inactive* phenotype classification was associated with donor risk factors, breast tissue composition, expression of other candidate genes, and gene signatures (Supplement Table 4). Other gene expression signatures scored included cAMP lipolysis, adipocyte browning, SASP, AST, IGF1, IGF1R, IFN, TGFβ, and CSR activities; all signatures, their gene components, and methods of score calculation are provided in Supplement Table 1. In addition, a numeric *Active/Inactive* signature score was calculated for correlation with other batch-specific numeric sample characteristics as a sign-corrected average using the following mathematical formula where Ss = signature score for that sample, Tg = TPM of the gene, *D* = set of downregulated signature genes, and *U* = set of upregulated signature gene values as listed in Supplement Table 1:

$$ {S}_s=\frac{\sum_{g\in U}{\log}_2\left({\mathrm{T}}_g+1\right)-{\sum}_{g\in D}{\log}_2\left({\mathrm{T}}_g+1\right)}{\left|U\right|\left.+\left|D\right.\right|} $$

### Transcriptome expression of cancer gene hotspot mutations

Given the lack of available KTB germline sequence data (e.g., peripheral blood DNAseq), transcript-level BAM files were analyzed for potential somatic variants using a proprietary loci-based variant caller, Locus, against two curated lists of human cancer mutation hotspots: (i) the Memorial Sloan Kettering (MSK-IMPACT) list of actionable cancer targets [9] and (ii) the curated set of ~ 1760 cancer gene hotspot mutations recently found expressed in the bulk RNAseq data of ~ 6700 normal human tissue samples from ~ 500 different individuals donating to the Genotype-Tissue Expression (GTEx) project [10]. The lists of all cancer gene hotspot mutations/variants detected in the KTB RNAseq data present in both hotspot databases are provided in Supplement Table 2, and the heuristics used to graphically score these hotspot mutations in relation to other KTB sample characteristic included mutation likelihood score ≥ 5, variant allele frequency (AF) ≥ 0.02 and ≤ 0.40, variant allele read depth (AD) ≥ 2, and predicted non-silent amino acid (AA) change.

### Batch-specific phenotype assignment and batch-corrected transcriptome values associated with mammary adipocytes and fat metabolism

Table 1 shows the breast biopsy donor characteristics by sample batch, indicating that F and P batches were well balanced for donor age at biopsy, age at menarche, age at first birth, parity, BMI, family history, and Gail 5 year risk scores. After batch-specific classification of each sample’s transcriptome as either *Active* or *Inactive*, F and P batches demonstrated partially overlapping gene sets differentially expressed between their *Active* and *Inactive* phenotypic subsets (FDR *p* < 0.05): 7679 genes differed between the F batch phenotypes (Wilcoxon *p* = 1e−14), and 6614 genes differed between the P batch phenotypes (Wilcoxon *p* = 6.5e−08). However, in order to perform maximally powered gene set enrichment analysis (GSEA) and TumorMap analyses on the combined (F + P = 151) collection of *Active* (F = 47/96, P = 31/55) and *Inactive* (F = 49/96, P = 24/55) sample transcriptomes, we mapped all P transcriptome data into the F transcriptome RNAseq space using a quantile normalization procedure to minimize batch-specific gene expression differences [11]. This batch-correction approach used the F sample’s expression quantiles as the target distribution and the P sample’s expression quantiles as the source distribution, and this quantile normalization was performed separately for each HUGO gene, excluding zero expression genes from both source and target datasets (attaching them after the mapping was complete) so that both source and target quantiles were computed using only expressed gene values. GSEA and TumorMap analyses were then performed on the combined batch-integrated gene expression dataset across 151 samples after each sample was phenotyped within its batch as either *Active* or *Inactive*.

**Table 1:** Donor characteristics by breast sample batch

Characteristic	F batch	P batch	*p* value^a^
	*N* = 96	*N* = 55	
Age at biopsy (years)			.73
Mean	44.75	45.36	
Age at menarche (years)			.73
Mean	12.57	12.49	
Parity (live births)			.21
Mean	2.18	2.00	
Age at first birth (years)			.04
Mean	27.56	25.82	
BMI (kg/m^2^)			.31
Mean	30.32	28.87	
Family history^b^			.76
Percent positive	25.0%	23.62%	
Gail 5 year risk scores			.51
Mean	1.34%	1.17%	

^a^*p* value is the chi-squared *p* value for difference in means between samples

^b^At least one first-degree relative with breast cancer

GSEA (https://www.gsea-msigdb.org/gsea/index.jsp; v2.2.4) was used to identify gene sets upregulated within the *Active* samples. A compendium of 18,408 gene sets was assessed for enrichment, including gene sets and pathways collected from a variety of sources (GO, HumanCyc, IOB, MSigdb, NCI, NetPath, Panther, Reactome, WikiPathways, and KEGG). The batch-corrected expression matrix from the KTB samples was used to compare the *Active* with the *Inactive* phenotypes, and 1000 phenotype permutations were used. Adipose-associated gene sets were identified by filtering gene set names with “fat,” “adip,” and “lip” and then removing names with terms such as “sulfate” and “sulfation,” resulting in 505 (2.7%) adipose-associated gene sets from the full set of 18,408. Gene sets enriched at FDR ≤ 10% were identified (Supplement Table 3), supporting further hypothesis-driven comparison of the *Active* transcriptome phenotype for mammary adipocyte characteristics and fat-specific metabolism pathways. Those expression signatures interrogated included cAMP-dependent adipocyte lipolysis [8, 12], white adipose tissue browning [13], senescence-associated secretory phenotype (SASP), and autophagy-to-senescence transition (AST) as found associated with aging mammary tissue [14], IGF1R and IGF1 ligand activation signatures [15, 16], and immune/inflammatory modules (IFN, TGFB, CSR) previously associated with malignant breast tissue [17, 18]. Single gene transcripts interrogated include those associated with varying adipocyte functions (FGF2, IGF1, CAP1, BNIP3, CAV1, LEP, LEPR, ADIPOQ, IKBKG, CCL13, SERPIN1) as well as general mesenchymal (CD38, CD68, IL6, SERPINE1, WIF1, IGF1R) and epithelial (KIT, MYB, TRPS1) cell states.

### TumorMap visualization of *Active* and *Inactive* normal breast transcriptomes and attribute mapping

As recently described and frequently used to compare transcriptome similarities and differences between tumor types and/or their molecular subtypes, the TumorMap is an interactive website tool enabling visualization of multi-dimensional patient sample data in a two-dimensional layout [19]. Here we used TumorMap to compare both batch-specific and batch-integrated transcriptome layouts for our 151 KTB normal breast samples, related these to 1096 TCGA-determined breast cancer transcriptomes across their five different molecular subtypes [20], and overlayed the map with KTB normal breast attributes. A sample-by-sample similarity matrix was first computed from the sample-by-gene mRNA expression matrix. The TumorMap’s force-directed layout engine then used the similarity matrix to position all samples in a two-dimensional space; samples that are close to each other in the multi-dimensional RNAseq space are also close to each other in the two-dimensional TumorMap layout. Locality patterns in the distribution of various sample attributes are revealed by coloring the samples in the layout based on their attribute scores; this form of spatial correlation analysis helps find pattern associations between attributes (e.g., co-occurring or mutually exclusive pairs) not easily found by direct sample correlations.

### Statistics

RNAseq transcriptome data, derived as described earlier, were analyzed using Bioconductor R (www.bioconductor.org) software programs, and normalized RSEM TPM values were used to compare batch-specific single gene expression levels and multi-gene signature scores, the latter calculated as described above. Histologic and gene expression co-variates subjected to statistical association for all batch (*F*, *P*) and *Active/Inactive* classified KTB samples are summarized in Supplement Table 4. Gail 5-year risk scores were calculated using the Breast Cancer Risk Assessment Tool (https://bcrisktool.cancer.gov). All tabulated data (Supplement Table 4) were compared for median and mean value differences between sample batches and according to their *Active/Inactive* classifications by chi-square and *T* test. All graphical plots show single, median or mean (± SD) values as described; grouped measures (e.g., box-whisker plots) were statistically compared by Wilcoxon rank-sum test and correlations evaluated by Pearson’s linear regression (Rp) and/or Spearman (Rs) analyses. Significant differences were determined as *p < 0.05 or **p < 0.01.

## Results

**Fig. 1:**
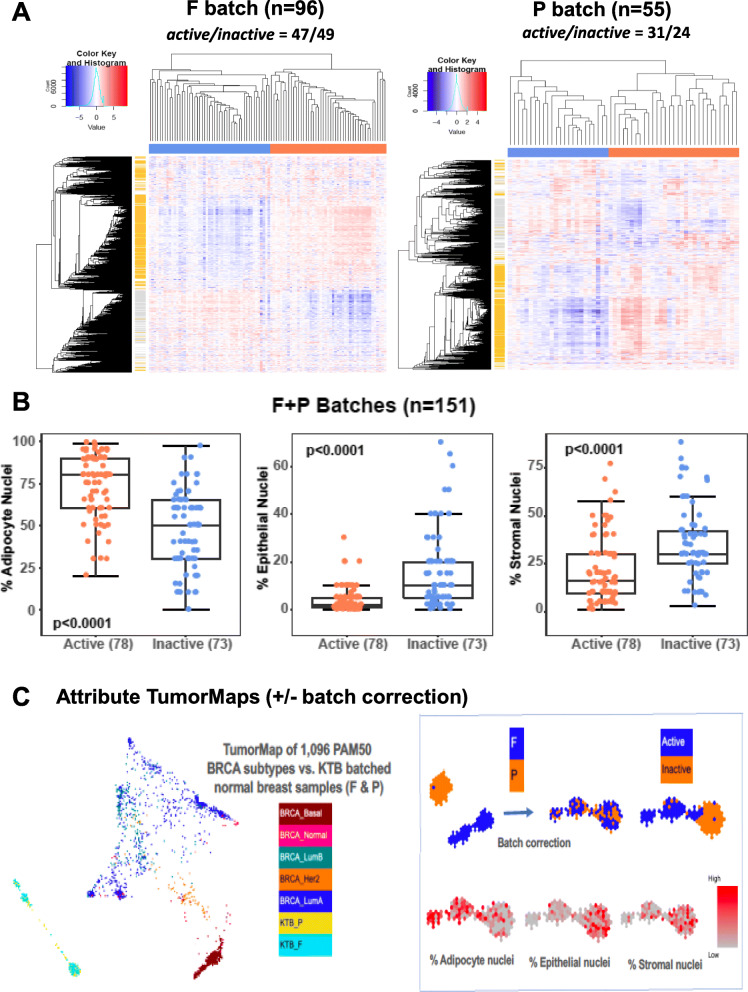
Classification of normal breast transcriptomes into *Active* and *Inactive* phenotypes and increased adipocyte association with *Active* samples. **a** Unsupervised clustering heat map (red = increased gene expression, blue = decreased gene expression) showing batch-specific assignment of *Active* (orange covariate bar) and *Inactive* (blue co-variate bar) phenotype samples. Horizontal dendrogram = samples, vertical dendrogram = 1318 classifying genes (gold bars = genes upregulated for *Active*, gray bars = genes upregulated for *Inactive* phenotype). **b** Box plots of normal breast cell composition (% adipocyte, stromal, epithelial nuclei) across all 151 samples relative to their transcriptome phenotype assignments (expression cluster *Active* or Inactive). **c** Left panel: two-dimensional TumorMap layouts [19] of all KTB normal breast full transcriptomes after batch-integration and projected relative to 1096 TCGA breast cancer full transcriptomes. Batch-integrated normal breast transcriptome samples colored according to batch (blue = *F*, yellow = *P*), and TCGA breast cancer transcriptomes colored according to their PAM50 subtype [20]. Right panel: normal breast full transcriptome TumorMaps presented in isolation before and after batch-correction, showing good sample set integration of both F and P batch samples and preserved spatial separation of *Active* and *Inactive* phenotypes, and overlay of the integrated set with color scores (red = high) of their individual cell compositions (% nuclei)

Unsupervised clustering of the 96 F and 55 P batch healthy breast RNAseq transcriptomes using their 1318 most variably expressed genes from the previously validated *Active/Inactive* multi-gene classifying signature (Supplement Tables 1; 3, 4) yielded the two normal breast heat maps shown in Fig. 1a; these two heat maps identified 47/96 F batch samples and 31/55 P batch breast samples for a total of 52% of all KTB samples as having the *Active* transcriptome phenotype. Batch-specific and combined cohort (F + P) box-whisker plots show that the *Active* samples possess 1.6-fold more (~ 80%, Wilcoxon *p* = 3.9e−11) adipocyte nuclei and 0.5-fold fewer stromal (Wilcoxon *p* = 4.3e−07) and epithelial (Wilcoxon *p* = 1.2e−10) cell nuclei (Fig. 1b). KTB donors with the *Active* phenotype had no difference in BMI but significantly higher Gail scores (1.46 vs. 1.18; *p* = 0.007).

After using a quantile normalization batch-correction procedure to integrate the two sets of normal breast transcriptome values, we used a two-dimensional visualization tool, TumorMap [19], to compare both the batch-specific and batch-integrated normal breast transcriptional landscapes to that of 1096 TCGA breast cancer samples spatially separated into their five different intrinsic subtypes (Fig. 1c): LumA, LumB, Her2, Normal-like, and Basal [20]. By generating isolated TumorMaps of the KTB normal breast transcriptomes we could superimpose individual attributes onto each sample including batch identity (F, P), *Active/Inactive* phenotype assignment, percent cell type composition (adipocyte, stromal, epithelial nuclei), and various single or multi-gene expression scores. From these attribute maps we see that the integrated normal breast transcriptional landscape still preserves the spatial distinction between *Active* and *Inactive* phenotype samples; as well, they serve to visually illustrate enrichment of the *Active* samples with higher adipocyte content and *Inactive* samples with higher stromal and epithelial cell content (Fig. 1c). Likewise, microscopically determined TDLU scores or mRNA expression of epithelial-specific genes like KIT and TRPS1 are seen to map preferentially over those normal breast samples possessing the highest epithelial content (Supplement Figure 1). In contrast, while macrophage and CD68 gene module signatures indicate that these immune attributes associate best with adipocyte content, a CD8 T cell immune signature preferentially associates with higher epithelial content samples (Supplement Figure 2). The numeric correlation coefficients and *p* values supporting these attribute maps, as well as all other study covariates for each sample, are summarized in Supplement Table 4.

**Fig. 2:**
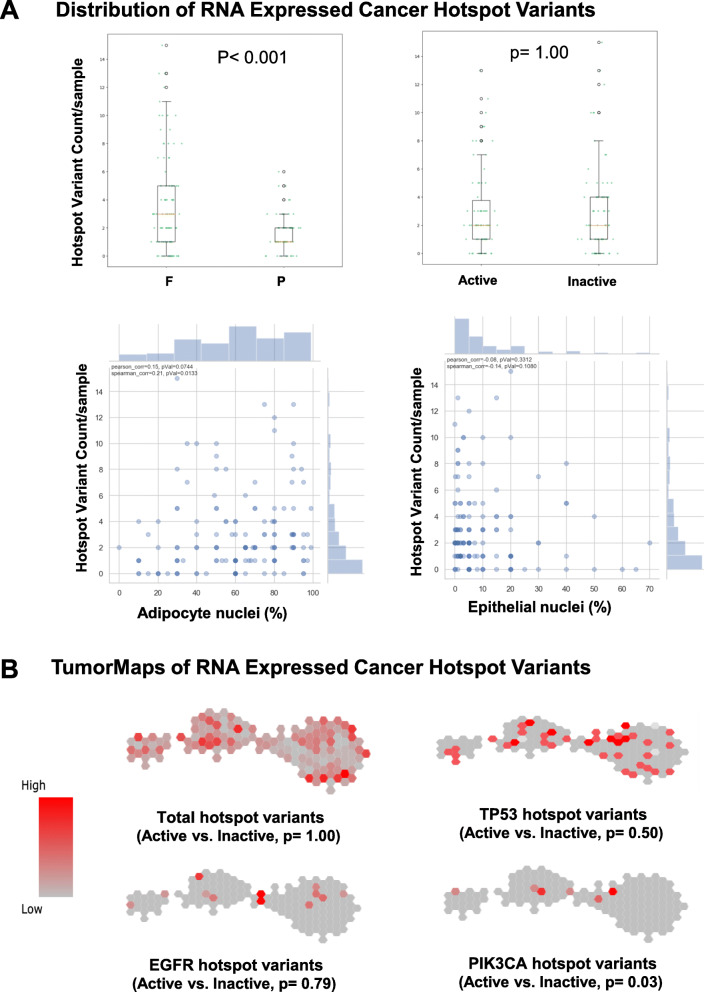
Normal breast sample scatterplots and TumorMaps of their transcriptome-expressed cancer gene hotspot mutations. As described in the “Methods” section (and detailed in Supplement Table 2), frequencies of the filtered list of detected 567 cancer gene hotspot mutations (variant counts/sample) are plotted: **a** according to sample batch (*F*, *P*) and prior transcriptome assignment as either *Active* or *Inactive*, and relative to each sample’s adipocyte or epithelial content (% nuclei) and **b** onto batch-integrated transcriptome TumorMaps with each sample overlaid by its relative hotspot variant frequency score (red = high) and mapped according to the cancer gene identity (total, TP53, EGFR, or PIK3CA variants)

Transcript-level BAM files within each batch were also analyzed independently and in depth to identify the expression of > 5140 low-frequency gene loci variants across a curated (MSK-IMPACT) list of actionable cancer targets [9], recording the variant nucleotide mutation, a calculated likelihood score for the rarely expressed variant, total read depths at both the reference and variant allele sites, and the resulting variant allele frequency (Supplement Table 2). These variant calls were then filtered against an independent listing of ~ 1760 cancer gene hotspot mutations identified by RNAseq analysis as being expressed in ~ 6700 normal tissue samples donated to the GTEx project, including 180 different normal breast samples [10]. This filtered call list of 567 hotspot mutations, subjected to the heuristics described in the “Methods” section, thus represents a conservative identification of low-frequency cancer gene mutation events expressed in our combined cohort of KTB normal breast samples: 464 in F batch and 103 in P batch samples (Supplement Table 2). Despite the significantly different number of mutation calls in F and P batch samples, the cancer gene hotspot mutation burden (counts/sample) was not significantly different between *Active* and *Inactive* normal breast samples (Fig. 2a), but there appeared to be a weakly positive correlation between this mutation burden and sample adipocyte content (Spearman *r* = + 0.21, *p* = 0.01), and insignificant negative correlation between mutation burden and sample epithelial content (Spearman *r* = − 0.14, *p* = 0.11). As previously reported in the GTEx set of human breast tissue samples [10], TP53 hotspot mutations occurred most frequently; EGFR and PIK3CA mutations were also detected, and, of these two epithelium-enriched mutation events, only the infrequent PIK3CA hotspot mutations showed any statistical association with the *Active* phenotype (Fig. 2b, *p* = 0.03).

GSEA performed on the batch-integrated (F + P) transcriptome values identified 186 gene sets upregulated in the *Active* set compared to the *Inactive* set, at FDR ≤ 10% (Supplemental Table 3). Of these, 21 (11.2%) were adipose-associated pathways, including “TRANSCRIPTIONAL REGULATION OF WHITE ADIPOCYTE DIFFERENTIATION” (from Reactome) and “HALLMARK_ADIPOGENESIS” (from MSigDB) which showed the second and third lowest FDR. Of the total 18,408 gene sets in the compendium, 505 are adipose-associated (2.7%), suggesting that adipose-associated gene sets are well represented among those gene sets significantly upregulated in the *Active* relative to *Inactive* samples.

To orthogonally investigate this GSEA observed association of *Active* samples with differentially expressed fat pathways, the top 200 upregulated genes used to classify the *Active* transcriptome phenotype were sent to Human Cell Atlas investigators to interrogate two reduction mammoplasty samples freshly disaggregated into discrete epithelial, fibroblast, adipocyte, endothelial, and immune/inflammatory cell fractions and subjected to 10× single-cell RNAseq (scRNAseq) analysis, as previously described [21]. These investigators reported that only the mammary adipocyte fractions from the reduction mammoplasty samples showed above baseline expression with varying degrees of overexpression of the 200 most upregulated *Active* signature genes, signifying that these *Active* signature genes primarily report on altered mammary adipocyte expression (personal communication, Kai Kessenbrock, Ph.D., kai.kessenbrock@uci.edu). Given both the GSEA and scRNAseq findings, as well as the significantly increased number of adipocytes enumerated in the *Active* phenotype samples, further microscopic and gene-specific analyses of the KTB samples were undertaken to compare potential differences in adipocyte morphology and fat metabolizing pathways between the *Active* and *Inactive* samples.

**Fig. 3:**
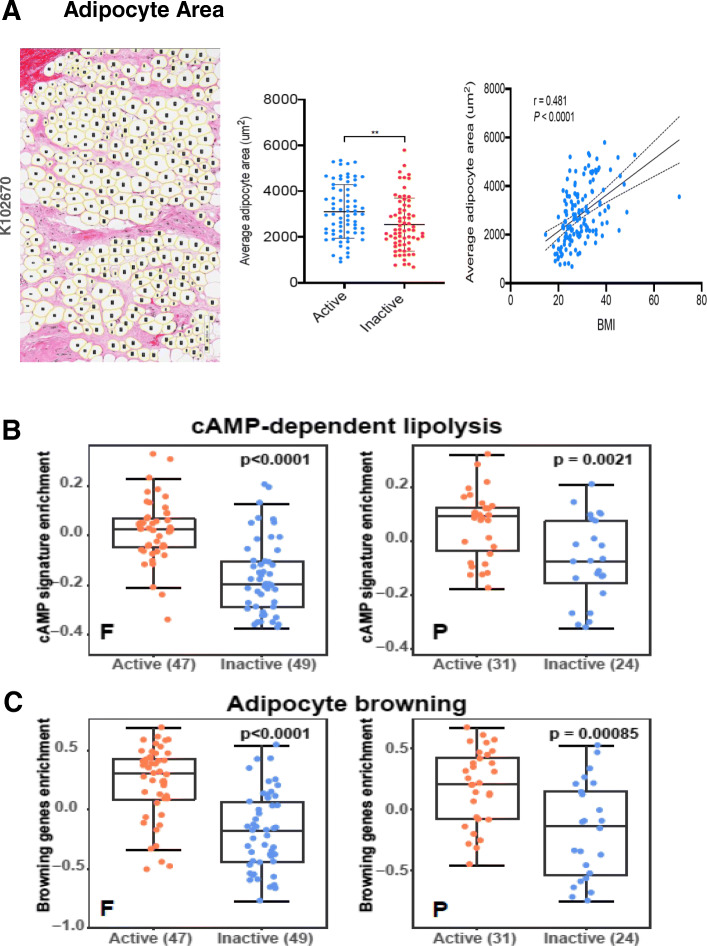
Normal breast adipocyte size and gene expression signatures in relation to *Active* and *Inactive* transcriptome phenotypes. **a** Representative H&E image (sample K102670) showing automated adipocyte delineation for cross-sectional area determination on each sample, and distribution plots of mean adipocyte areas for all 151 samples according to their donor’s BMI (*r* = 0.48, *p* < 0.0001) and the sample’s transcriptome phenotype as either *Active* or *Inactive* (**p < 0.01). **b** Batch-specific (F, P) box plot distributions of a previously reported breast cancer-associated cAMP-dependent lipolysis signature [8, 12] according to sample transcriptome phenotype assignment (expression cluster) as either *Active* or *Inactive*. **c** Batch-specific (*F*, *P*) box plot distributions of a previously reported breast cancer-associated white adipocyte browning signature [13] shown according to sample transcriptome phenotype assignment (expression cluster) as either *Active* or *Inactive*

**Fig. 4:**
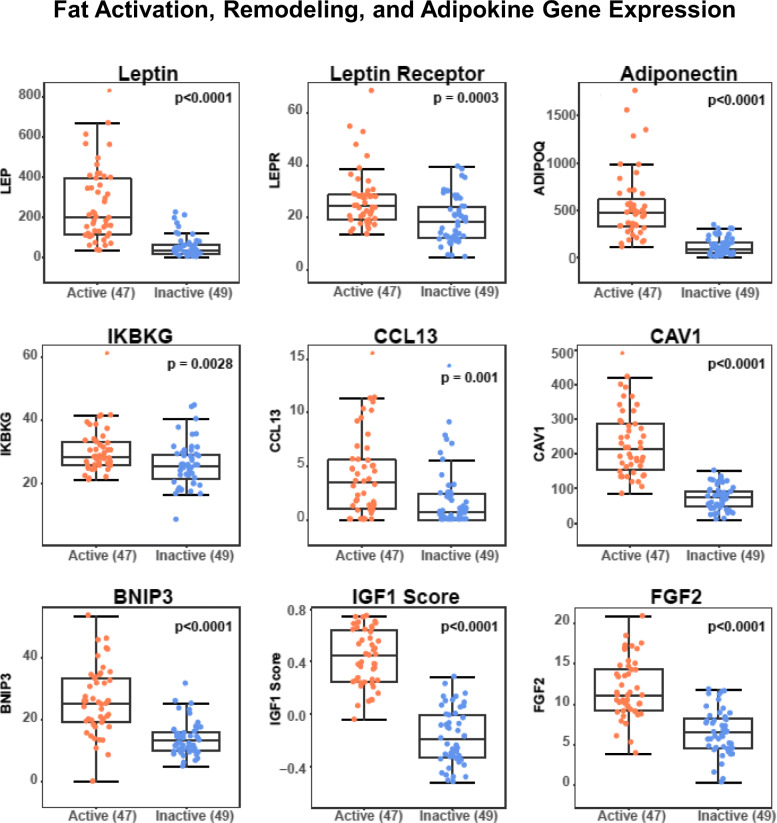
Normal breast expression of genes representing fat activation, remodeling, pro-inflammatory signaling, and growth factor expression. F batch-specific box plot distributions of single genes reflecting adipocyte activation (leptin, leptin receptor, adiponectin), adipocyte pro-inflammatory signaling (IKBKG, CCL13), remodeling (CAV1, BNIP3), and adipokine growth factor expression (IGF1 score, FGF2) shown according to sample transcriptome phenotype assignment, *Active* or *Inactive*. Comparable P batch-specific box plot distributions are shown in Supplement Figure 3

**Fig. 5:**
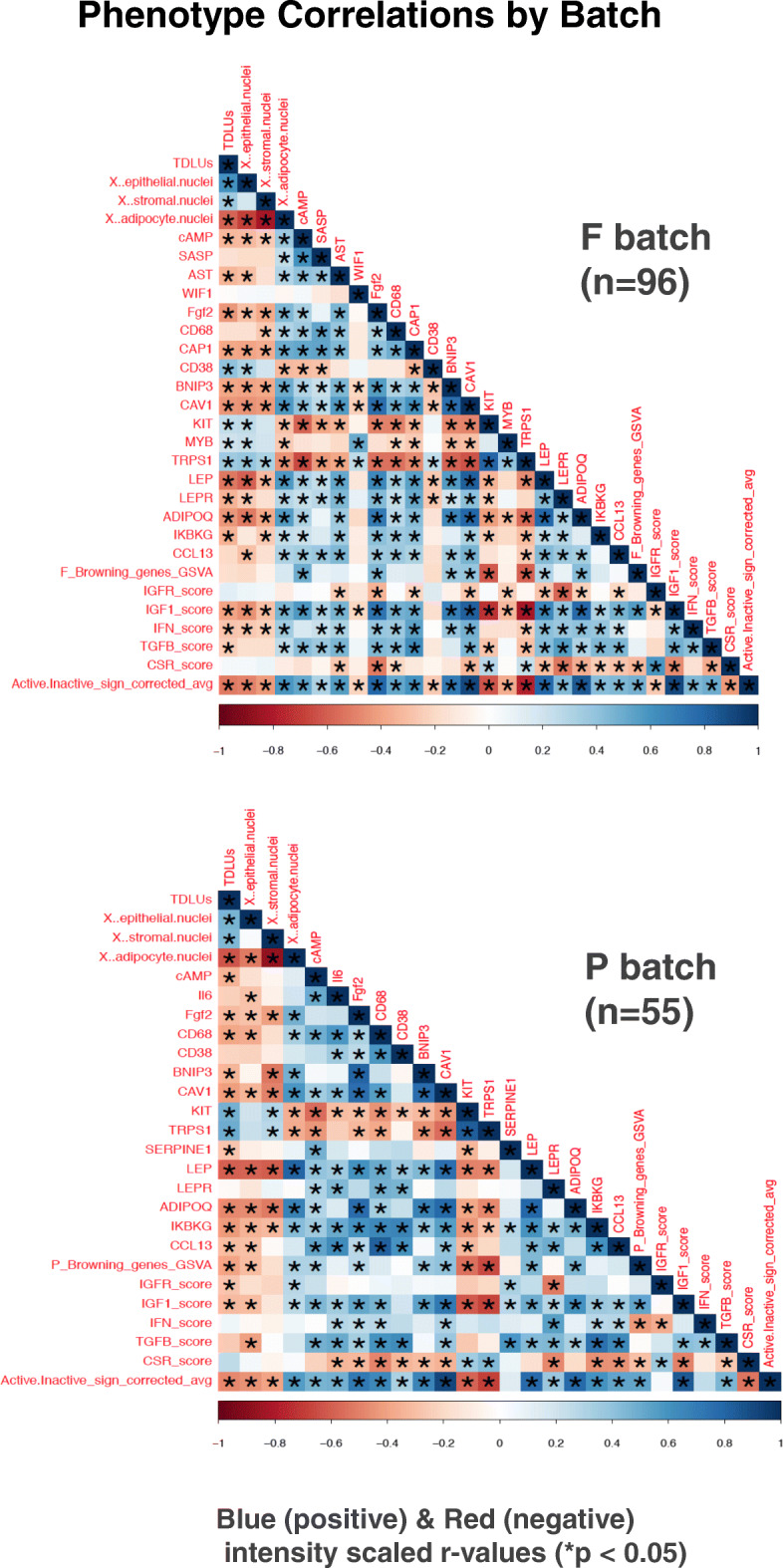
Batch-specific phenotype correlations summarizing all normal breast sample microscopic features and their measured gene expression relationships. Matrices show Pearson correlation heatmap relationships between all histologic, single gene and gene signature expression values, determined separately on the F (*n* = 96) and P (*n* = 55) batch normal breast samples, with correlations shown as blue (positive) or red (negative) intensity scaled *r* values (− 1 to + 1) and asterisks signifying *p* < 0.05 significance

Mean adipocyte cross-sectional areas were determined by automated analysis of each sample’s H&E slide, as described in the “Methods” section and previously reported [8], with adipocyte area delineation for a representative KTB sample slide illustrated in Fig. 3a. The calculated mean adipocyte area (μm^2^) for each breast sample correlated with that donor’s BMI (*r* = 0.48, *p* < 0.0001), and normal breast samples classified as having the *Active* transcriptome phenotype showed significantly larger adipocytes (*p* < 0.01) than those with the *Inactive* phenotype (Fig. 3a). Multi-gene signatures corresponding to previously reported breast cancer-associated adipocyte changes, including cAMP-dependent lipolysis [8, 12] and white adipose tissue browning [13], were also significantly elevated (Wilcoxon *p* ≤ 0.002) in the *Active* samples relative to the *Inactive* samples within both F and P batches (Fig. 3b, c). Likewise, expression of single genes associated with adipocyte activation (leptin, leptin receptor, adiponectin), pro-inflammatory fat signaling (IKBKG, CCL13), fat remodeling (CAV1, BNIP3), and adipokine growth factors (IGF-1, FGF2) were all significantly elevated in the *Active* samples relative to the *Inactive* samples within both batches (Fig. 4 for F batch results, Supplement Figure 3 for P batch results). Summary representations of all microscopic features and gene expression continuous values, and their correlations with one another across all samples within each batch, are shown in Fig. 5. All numeric correlation coefficients and *p* values supporting these (Fig. 5) co-variate associations are provided in Supplement Table 4.

## Discussion

In this KTB cohort of predominantly non-obese Caucasian women without any current or past history of breast disease (characterized in Tables 1 and 2), more than half were found to possess the same *Active* mammary transcriptome phenotype previously shown to characterize the peritumor microenvironment of ~ 40% of newly diagnosed breast cancers associated with a near 3-fold excess risk of later-life breast cancer mortality [3, 4]. While > 80% of our donor cohort possessed Gail scores less than 1.65 and thus not recommended for breast cancer chemoprevention, those with *Active* transcriptomes possessed significantly higher Gail scores relative to those without, supporting their greater future risk for developing breast cancer (Table 2; Supplement Table 4). For this KTB cohort, however, at least another decade of follow-up will be necessary to prospectively test this predicted outcome. In the interim, the finding of a risk-associated normal breast *Active* transcriptome phenotype evident before any histologic evidence of breast neoplasia offers a unique opportunity to discern the composition and molecular nature of a potentially pro-tumorigenic breast microenvironment.

**Table 2:** Donor characteristics by breast sample phenotype

Characteristic	Active	Inactive	*p* value^a^
	*N* = 78	*N* = 73	
Age at biopsy (years)			.01
Mean	47.13	42.25	
Age at menarche (years)			.06
Mean	12.78	12.32	
Parity (live births)			.13
Mean	2.01	2.22	
Age at first birth (years)			.95
Mean	26.89	26.92	
BMI (kg/m^2^)			.75
Mean	29.80	29.41	
Family history^b^			.54
Percent positive	25.6%	23.3%	
Gail 5 year risk scores			.01
Mean	1.46%	1.18%	

^a^*p* value is the chi-squared *p* value for difference in means between samples

^b^At least one first-degree relative with breast cancer

The recent TCGA study confirming a 3-fold excess 10-year survival risk associated with the cancer-adjacent *Active* phenotype revealed that this multi-gene expression signature is closely mirrored by a microRNA (miR) signature composed of > 300 different miRs, including some associated with breast cancer and several (e.g., miR-200 members) known to directly regulate epithelial-to-mesenchymal transition (EMT), angiogenesis, and metastasis [4]. As well, that TCGA study demonstrated that both *Active* miR and mRNA classifying signatures correlated strongly with increased adipocyte content [4], in keeping with the current study’s microscopic and transcriptomic GSEA findings as well as the independent scRNAseq findings by Human Cell Atlas investigators that our *Active* gene classifying signature reports selectively on the variable expression of adipocytes as isolated from reduction mammoplasty samples (personal communication, K. Kessenbrock).

While the transcriptomes of normal breast samples from cancer-adjacent and reduction mammoplasty samples have previously been shown to be distinguishable [1], this study of KTB donated biopsy samples from women without any breast disease illustrates the potential introduction of technical batch artifacts when samples are differentially processed and fixed prior to RNA extraction and RNAseq assessment, although such artifacts may be largely corrected using advanced batch-integration algorithms as employed here. Fortunately, we observed excellent F-to-P batch concordance between patient and sample characteristics (Tables 1 and 2), and for almost all gene-gene and gene-attribute correlations (Figs. 3, 4, and 5). Using the batch-integrated transcriptome values and a new two-dimensional visualization tool, TumorMap [19], we could spatially integrate and map all the samples into a common transcriptional landscape while still preserving their spatial differentiation between *Active* and *Inactive* phenotypes (Fig. 1c). This visualization tool also illustrated the marked distinction between expression profiles of fat and stromal cell predominant normal breast samples relative to the transcriptional landscape of > 1000 different epithelium-enriched (> 60%) TCGA breast cancers representing all five intrinsic/PAM50 subtypes (Fig. 1c). Overlying various KTB sample attributes onto the integrated normal breast transcriptome landscape enabled sample-by-sample visual maps of how the *Active* normal breast samples are more enriched with adipocytes while the *Inactive* samples are more enriched in stromal and epithelial cells (Fig. 1c). Confirmed by histological scoring (Fig. 1b), the *Active* KTB normal samples possessed 1.6-fold more (80% vs. 50% mean nuclei, *p* = 3.9e−11) adipocytes and 0.5-fold fewer stromal and epithelial cells (*p* < 1e−6).

Prior studies have shown that normal female mammary composition can vary from < 10 to > 70% adipocytes, and, despite the acknowledged absolute dependence of normal mammary gland development on a mature mammary fat pad, there remains poor understanding behind the marked variations observed in mammary gland white adipose tissue (WAT) content across premenopausal females as well as its weak and inconsistent positive association with female age and obesity [22–24]. Notable here, neither age nor BMI scores were significantly different between KTB donors with *Active* vs. *Inactive* normal breast phenotypes (Table 2, Supplement Table 4). Unexpectedly, our 151 samples from parous and predominantly peri- or post-menopausal women showed no consistent evidence for an aging effect on their composite breast tissue as assessed by gene signatures specific for DNA damage response, cellular senescence, senescence-associated secretory profile, or autophagy-to-senescence transition, although our KTB samples possessed a minority (< 50%) of stromal cells whereas earlier studies describing these aging signatures specifically interrogated breast stroma excluding adipocytes [14].

Microscopic evaluation of our KTB tissue sections detected only rare leukocyte infiltrates and no crown-like structures within these adipocyte-rich normal breast samples (Supplement Table 4), yet the RNA from multiple adjacent thick sections revealed immune/inflammatory cell signatures [17, 18] suggesting (i) more CD68+ macrophages mapping onto the TGFβ+, adipocyte-rich *Active* samples and (ii) more CD8+ T cells mapping onto CSR+, KIT+, and TRPS1+ epithelial- and stromal-rich *Inactive* samples (Fig. 5c, Supplement Figures 1 and 2, Supplement Table 4). In addition to their quantitative excess of adipocytes, *Active* normal breast sample signatures showed greater TGFβ expression and more M2 tumor-promoting (CD68+) macrophages associating with their hypertrophied mature adipocytes (Fig. 3a), a previously described feature of post-weaning early-stage mammary involution [24]. The observed upregulation of NOD signaling intermediates, IKBKG and CCL13 (Fig. 4), is also consistent with a pro-inflammatory adipocyte population within these *Active* breast samples [25]. WAT hypertrophy in association with pro-inflammatory adipocyte signaling characterizes the obesity-driven microenvironment thought to commonly promote breast, endometrial, prostate, and gastrointestinal cancers [26–29]. While adipose tissue-induced inflammation in the absence of cancer is most typically a feature of visceral WAT, mammary gland WAT inflammation has been proposed as a breast cancer risk factor [30]. Although others believe that the adipokines hormonally secreted by hypertrophic visceral WAT are the transforming mediators between truncal obesity and epithelial malignancies like breast cancer [31, 32], it is relevant to note that imaging studies now indicate that > 90% of all breast cancers, unlike benign breast lesions, arise at the fat-gland interface [33].

By their hypertrophic feature alone, *Active* normal breast adipocytes appear akin to visceral WAT but distinct from what have been called cancer-associated adipocytes, which are typically smaller, more spindly, and show gap junction connections to adjacent malignant breast epithelium—the latter enabling lipolytic depletion of triglyceride stores to fuel tumor mitochondria by supplying free fatty acids and metabolites for malignant cell growth and progression [8, 27]. On the other hand, the hypertrophied adipocytes spanning the small islands of normal breast epithelium in our KTB *Active* samples exhibit a dysregulated transcriptional pattern quite typical of cancer-associated adipocytes [8, 13, 27–29], including increased expression of leptin (and its receptor) and adiponectin (Fig. 4), lipoprotein digesting and adipocyte remodeling genes like CAV1 and BNIP3 (Fig. 4), expression signatures defining increased cAMP-dependent lipolysis (Fig. 3b) and adipocyte “browning” (Fig. 3c), and multi-fold overexpression of the potent breast tumor-promoting adipokine growth factors, IGF-1 and FGF2 (Fig. 4). Leptin, produced mainly by adipocytes, has been proposed as a mediator between obesity, inflammation, and breast cancer development [34]. IGF-1, produced by adipocytes and essential for normal mammary gland growth and development, has also been implicated as a mechanistic link between obesity and breast cancer development [35–37]. In addition to the chemo-attractant and macrophage-recruiting properties of other adipokines, adiponectin has been shown to cross-talk with the IGF-1 axis and thereby potentiate its growth-promoting effect on breast cancer cells [38]. Perhaps the most potent of adipokines capable of initiating and promoting malignant transformation of breast epithelium is FGF2, essential for normal mammary gland development and stem cell function [39] yet a proven driver of various cancers and a target for new anti-cancer therapeutics [40], experimentally capable of inducing both skin and mammary epithelial cancers [31, 32].

The above-described properties of pro-tumorigenic adipokine growth factors may either mutationally initiate breast tumorigenesis or simply promote the growth of an already initiated but microscopically occult population of pre-neoplastic breast epithelial cells, much like the well-described properties of cancer-associated fibroblasts [41]. A recent deep RNAseq study of normal GTEx organs and tissues has revealed that, despite their healthy histologic appearance, a significant proportion of the 180 different normal breast samples in that collection actually expressed from 1 to 30 cancer-driving gene mutations per sample, the sample’s overall mutational burden dependent in part on the donor’s age but with many of the expressed mutations occurring within proven cancer gene hotspots [10]. Our KTB normal breast RNAseq study supports this surprising albeit infrequent finding of expressed cancer gene hotspot mutations (Fig. 2, Supplement Table 2), and while we did not observe any difference in overall mutation burden or frequency between the *Active* and *Inactive* normal breast samples, further studies are needed to confirm our preliminary observations that normal breast mutation burden may correlate with adipocyte content and that *Active* samples may indeed possess more PIK3CA hotspot mutations.

## Conclusions

In summary, our findings of a risk-associated *Active* transcriptome linked to an activated adipocyte population, along with infrequent but detectable cancer gene hotspot mutations, all expressed in histologically normal-appearing adult breast tissue, suggest that a pro-tumorigenic adipocyte microenvironment can not only pre-exist the development of breast neoplasia but also provide fertile soil for a newly seeded, mutated and replication-competent mammary epithelial cell, whose growth is further promoted toward the development of a clinical breast cancer. Moving forward, breakthrough advances in personalized breast cancer risk assessment and prevention strategies would seem to depend on being able to screen mature women for both their normal breast tissue mutational burden as well as the presence of a pro-tumorigenic microenvironment that includes activated mammary adipocytes.

## Supplementary information

**Additional file 1: Supplement Figure 1.** Isolated TumorMap of the batch-integrated normal breast transcriptomes overlain with color-scaled intensity scores (red = high) for their various epithelial attributes including % epithelial nuclei, TDLU scores, KIT and TRPS1 gene expression levels. All numeric sample scores can be found in Supplement Table 4. **Supplement Figure 2.** Isolated TumorMap of the batch-integrated normal breast transcriptomes overlain with color-scaled intensity scores (red = high) for their various tissue compositions (% stromal, adipocyte, epithelial nuclei) and transcriptome gene expression modules representing specific immune cell signatures (macrophage, CD68, CD8 Tcell). Modules are defined in Supplement Table 1 and their numeric signature scores are listed in Supplement Table 4. **Supplement Figure 3.** Normal breast expression of genes representing adipocyte activation, remodeling, and pro-inflammatory signaling for all P batch samples according to their sample transcriptome phenotype assignment as either *Active* or *Inactive* (as described in Fig. 4 legend).

**Additional file 2: Supplement Table 1.** Lists of genes included within each gene expression signature analyzed to determine *Active*/*Inactive*, cAMP lipolysis, adipocyte browning, SASP, AST, IGF1, IGF1R, IFN, TGFβ, and CSR activity scores. Also indicated is their reference sources and method of score calculation.

**Additional file 3: Supplement Table 2.** RNAseq cancer gene hotspot mutations detected in 151 KTB samples. Sheet 1 identifies the > 5100 unthresholded cancer mutation hotspot calls from the KTB RNAseq analysis occurring in the combined set of MSK-IMPACT curated cancer hotspot clinical targets [10] and the experimentally determined RNAseq identified set of expressed cancer gene hotspot mutations within > 6700 normal GTEx human tissue samples as recently described [11]. Sheet 2 (“normal_breast_rna.getz_list.092”) lists only those > 1760 cancer gene hotspot mutations from sheet 1 meeting the threshold mutation likelihood score > 5, while sheets 3 and 4 list the thresholded hotspot mutations according to F and P sample batches with each sample phenotyped as either *Active* or *Inactive*. Sheet 5 is a sample key mapping all KTB identification numbers, barcodes, and UUID numbers pertaining to the RNAseq results and cancer gene hotspot mutation calls. Of note, Fig. 2 panels (“Breast sample scatterplots and TumorMaps of RNA expressed cancer hotspot mutations”) include plots derived from the curated and thresholded hotspot mutations as listed in sheet 2.

**Additional file 4: Supplement Table 3.** Rank ordered GSEA (www.gsea-msigdb.org/gsea) analysis showing 186 gene sets upregulated (from total set of 18,408 gene sets) in the batch-integrated *Active* vs. *Inactive* transcriptome samples, at FDR ≤ 10%. Also shown are their individual nominal *p*-values, FDR q-values, their gene set size, and the 21 (11.2%) that are specifically involved in adipose-associated pathways.

**Additional file 5: Supplement Table 4**. Master spreadsheet listing showing sample and donor covariates for each of the 151 KTB barcodes including batch assignment (F or P), *Active/Inactive* phenotype assignment and score, donor features (including age, BMI, 5 year Gail risk scores), percentages of adipocyte/stromal/epithelial cell nuclei, TDLU counts, mean adipocyte areas, immune modules, and all gene signatures and single genes values used to calculate the Pearson correlations shown in Fig. 5.

## Data Availability

The primary transcriptome (RNAseq) data obtained on all tissue samples have been deposited on the public UCSC Xena functional genomics platform: https://xenabrowser.net/datapages/?cohort=Normal%20Breast%20(Benz%202020). All other primary data analyzed and presented in this study are located in the Supplementary tables attached to this manuscript.

## Abbreviations

AA: Amino acid
AD: Allele read depth
AF: Allele frequency
AST: Autophagy-to-senescence transition
BMI: Body mass index
ER: Estrogen receptor
FDR: False discovery rate
F batch: FFPE batch
FFPE: Formalin fixed, paraffin embedded
FGF2: Fibroblast growth factor-2
GSEA: Gene set enrichment analysis
GTEx: Genotype-Tissue Expression
H&E: Hematoxylin and eosin
IGF-1: Insulin-like growth factor-1
KTB: Komen Tissue Bank
mIR: MicroRNA
P batch: PFPE batch
PFPE: PAXgene fixed, paraffin embedded
RNAseq: Full transcriptome RNA sequencing
SASP: Senescence-associated secretory phenotype
TCGA: The Cancer Genome Atlas
TDLU: Terminal duct lobular unit
TPM: Transcripts per million
WAT: White adipose tissue
