# Supplementary material for: A risk-associated Active transcriptome phenotype expressed by histologically normal human breast tissue and linked to a pro-tumorigenic adipocyte population
Source: Breast Cancer Res. 2020 Jul 31;22:81. doi: 10.1186/s13058-020-01322-6 (PMC7395362; doi:10.1186/s13058-020-01322-6)
Supplement: Supplementary file 1 — Additional file 1: Supplement Figure 1. Isolated TumorMap of the batch-integrated normal breast transcriptomes overlain with color-scaled intensity scores (red = high) for their various epithelial attributes including % epithelial nuclei, TDLU scores, KIT and TRPS1 gene expression levels. All numeric sample scores can be found in Supplement Table 4. Supplement Figure 2. Isolated TumorMap of the batch-integrated normal breast transcriptomes overlain with color-scaled intensity scores (red = high) for their various tissue compositions (% stromal, adipocyte, epithelial nuclei) and transcriptome gene expression modules representing specific immune cell signatures (macrophage, CD68, CD8 Tcell). Modules are defined in Supplement Table 1 and their numeric signature scores are listed in Supplement Table 4. Supplement Figure 3. Normal breast expression of genes representing adipocyte activation, remodeling, and pro-inflammatory signaling for all P batch samples according to their sample transcriptome phenotype assignment as either Active or Inactive (as described in Fig. 4 legend). [file 13058_2020_1322_MOESM1_ESM.pptx]

## Slide 1
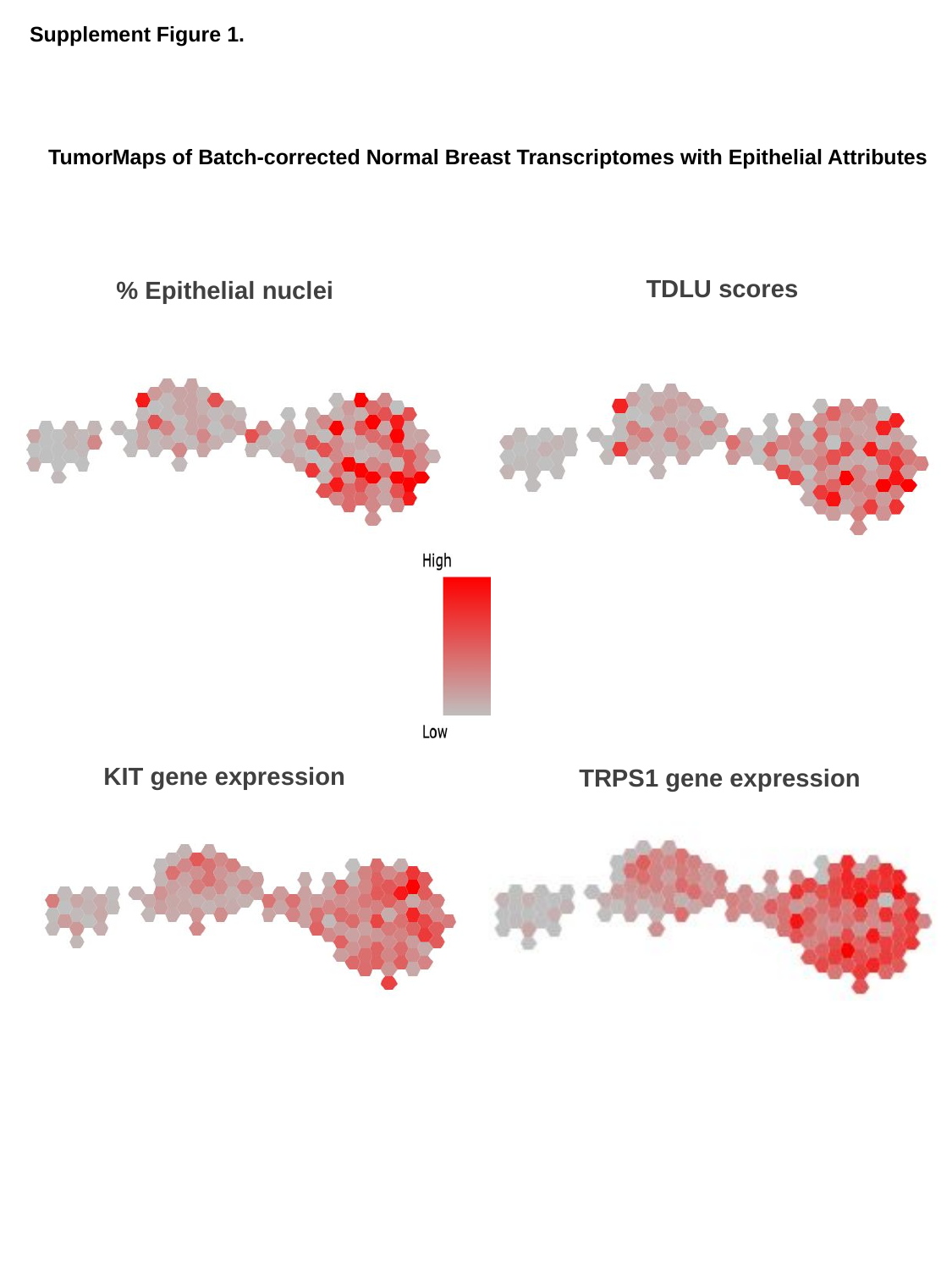

Supplement Figure 1.
TumorMaps of Batch-corrected Normal Breast Transcriptomes with Epithelial Attributes
TDLU scores
% Epithelial nuclei
KIT gene expression
TRPS1 gene expression

## Slide 2
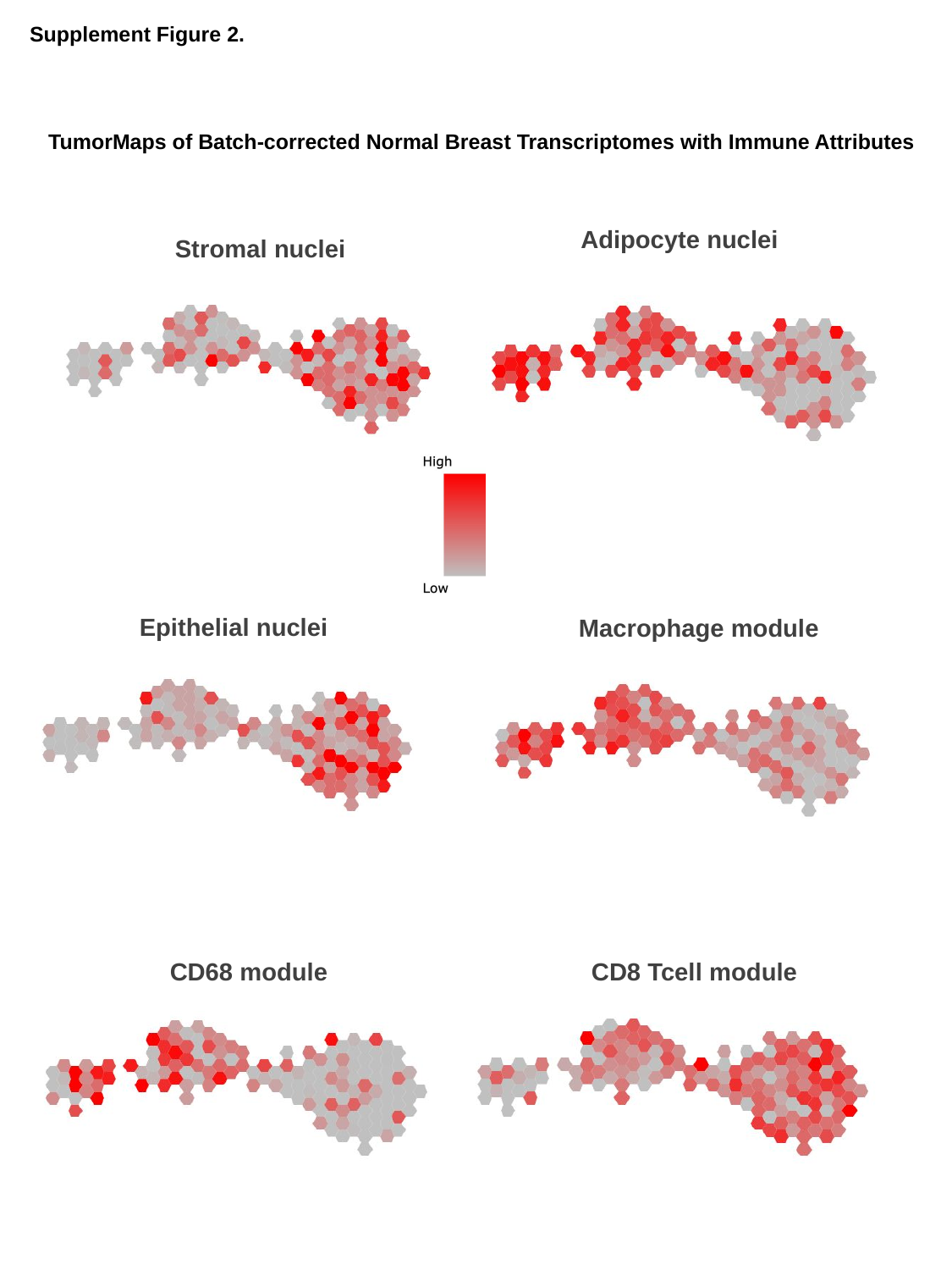

Supplement Figure 2.
TumorMaps of Batch-corrected Normal Breast Transcriptomes with Immune Attributes
Adipocyte nuclei
Stromal nuclei
Epithelial nuclei
Macrophage module
CD68 module
CD8 Tcell module

## Slide 3
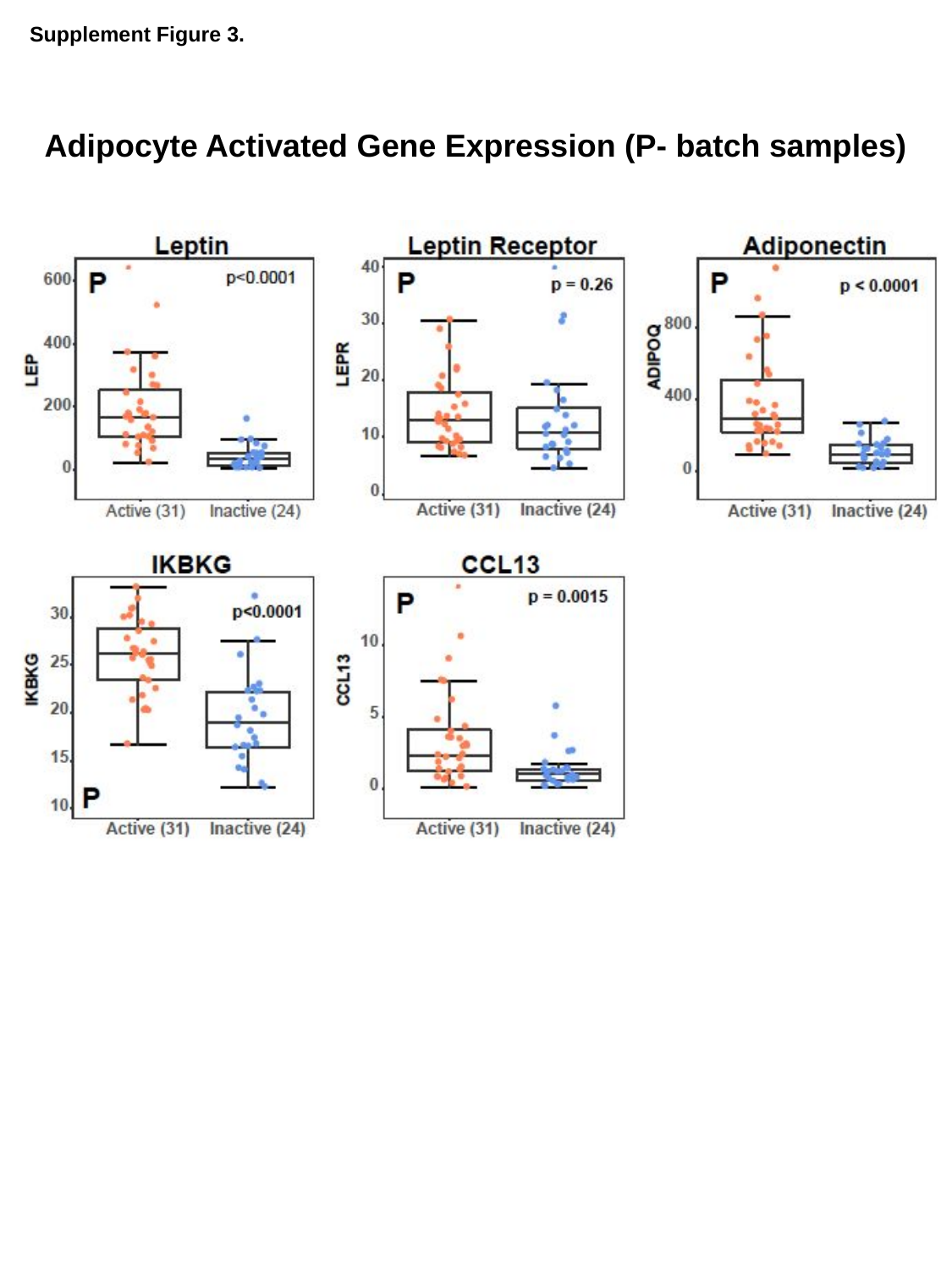

Supplement Figure 3.
 Adipocyte Activated Gene Expression (P- batch samples)
